# Pre-treatment ADC histogram-analysis at whole body diffusion-weighted MRI predicts disease free survival in ovarian cancer

**DOI:** 10.1186/1470-7330-15-S1-S5

**Published:** 2015-10-02

**Authors:** K Michielsen, I Vergote, F Amant, K Leunen, S Dymarkowski, F De Keyzer, V Vandecaveye

**Affiliations:** 1 Department of Radiology, Leuven Cancer Institute, University Hospitals Leuven, KU Leuven, Leuven, Belgium; 2 Department of Obstetrics and Gynaecology, Leuven Cancer Institute, University Hospitals Leuven, KU Leuven, Leuven, Belgium

## Aim

To prospectively evaluate the predictive value of pre-treatment histogram analysis of apparent diffusion coefficients (ADC) at whole body diffusion-weighted imaging (WB-DWI/MRI) for patient outcome in primary ovarian cancers.

## Methods

Institutional review board approval and informed consent were obtained for this prospective study. Forty-four women diagnosed with FIGO stage III or IV ovarian carcinoma underwent 3-Tesla WB-DWI/MRI using 2 b-values (b=0-1000 s/mm^2^), T2-weighted and contrast-enhanced T1-weighted sequences prior to treatment. The primary tumour was delineated using semi-automated software and was analysed by using an ADC histogram approach: mean and median ADC, standard deviation (SD), coefficient of variation (CoV, SD/mean), kurtosis and skewness were calculated. Kaplan-Meier with log-rank statistics were used to correlate baseline ADC parameters to disease free survival (DFS). Effects of confounding patients- and tumour-related factors were taken into consideration using Cox proportional hazard model.

## Results

5 patients underwent primary- and 39 interval debulking surgery completing 6 cycles of platinum-based chemotherapy. Survival analyses showed that lower CoV was associated with significantly longer DFS (median ± SD; 19±2 months for CoV<0.2601 versus 12±1 months for CoV>0.2601; p=0.002). After multivariable analysis, CoV remained an independent prognostic biomarker for DFS (p=0.003) when taking patient’s age, FIGO stage, tumour grade and cancer antigen (CA)-125 level into consideration as clinical prognostic factors.

## Conclusion

In this pilot study, pre-treatment ADC histogram analysis of primary ovarian cancer using the CoV was an independent predictive marker of DFS suggesting a correlation between tumour heterogeneity and treatment resistance. Further research should elucidate the correlation with overall survival.

